# Social epidemiology of multidimensional sleep health in early adolescence

**DOI:** 10.1038/s41390-025-04616-7

**Published:** 2025-11-28

**Authors:** Jason M. Nagata, Christiane K. Helmer, Isaac Frimpong, Keira Beltran Murillo, Alexander W. Heuer, Oliver H. Huang, Elizabeth J. Li, Colbey Ricklefs, Kyle T. Ganson, Alexander Testa, Jinbo He, Fiona C. Baker

**Affiliations:** 1 Department of Pediatrics, University of California, San Francisco, San Francisco, CA USA; 2 Factor-Inwentash Faculty of Social Work, University of Toronto, Toronto, ON Canada; 3 Department of Management, Policy and Community Health, University of Texas Health Science Center at Houston, Houston, TX USA; 4 Department of Biosciences and Bioinformatics, School of Science, Xi’an Jiaotong-Liverpool University, Suzhou, Jiangsu China; 5 Center for Health Sciences, SRI International, Menlo Park, CA USA

## Abstract

**Background:**

Poor sleep health is a significant concern in adolescents. This study examines the social epidemiology of sleep health in a large, diverse, national US sample of early adolescents.

**Methods:**

We analyzed cross-sectional data from Year 3 (2019–2021) of the US Adolescent Brain Cognitive Development Study to examine adjusted associations between sociodemographic factors and sleep duration, sleep efficiency (percentage of time asleep while in bed), chronotype (midpoint of sleep on free days), and social jet lag (difference in sleep onset between free and school days) using multiple linear regression models. Sleep metrics were derived from the Munich Chronotype Questionnaire. Interaction with race/ethnicity and sleep outcomes by household income and parental education was evaluated.

**Results:**

Among 10,082 adolescents (mean age 12.9 ± 0.7), average sleep duration was 8.9 (±1.5) hours, chronotype was 28.3 (midpoint 4:13 a.m.), and social jet lag was 2.3 h. Older age was associated with shorter sleep duration, later chronotype, and greater social jet lag. Gay/bisexual adolescents reported shorter sleep duration, later chronotype, and greater social jet lag. Black, Latino/Hispanic, and Native American adolescents generally had poorer sleep outcomes. Lower-income households were associated with shorter sleep duration and greater social jet lag. Black race and worse sleep outcomes were more strongly associated among higher-income/parental education households.

**Conclusion:**

Sociodemographic disparities in adolescent sleep call for targeted interventions to promote healthier sleep.

**Impact:**

- Sociodemographic disparities in adolescent sleep health outcomes have been studied, but few have explored associations with multidimensional sleep metrics.
- US early adolescents reported an average sleep duration of 8.9 h, with 50.5% of 11–12-year-olds and 61.0% of 13–14-year-olds meeting the American Academy of Sleep Medicine’s sleep guidelines.
- Older age, gay/bisexual, Black, Latino/Hispanic, Native American, lower household income, and lower parental education were associated with worse sleep outcomes, including shorter sleep duration, later chronotype, and greater social jet lag.
- The association between Black race and worse sleep outcomes was stronger among higher-income/parental education households.

## Introduction

Sleep is vital to the physical health, cognition, and emotional development of adolescents.^1,2^ However, adolescent sleep deprivation is a growing public health concern, with 57.8% of middle school students and 72.7% of high school students failing to meet recommended sleep guidelines.^3^ As adolescents gain independence, they are also more susceptible to delays in sleep regulation and circadian rhythm.^4^ Notably, research has found that inconsistencies in sleep timing and patterns are predictors of obesity, poor academic performance, and decreased cardiometabolic health.^5–8^

Two key aspects of adolescent sleep patterns are chronotype, which refers to an individual’s biologically influenced sleep-wake preference,^9^ and social jet lag, which refers to the misalignment between sleep patterns during school days and free days.^10^ Chronotype tends to shift later during adolescence, making it more difficult for older adolescents to align with early school schedules.^9^ This developmental shift contributes to a misalignment between internal circadian rhythms and external demands, which has been linked to difficulties in maintaining sufficient and consistent sleep, with downstream effects on mood, cognition, and overall well-being.^9^ Greater levels of social jet lag have been shown to predict lower cognitive performance and academic achievement, even after accounting for sociodemographic and sleep-related factors.^11^ These findings suggest that structural and psychosocial stressors may amplify circadian disruption.

Research highlights notable sleep disparities across racial, ethnic, and socioeconomic lines. Social epidemiology offers a framework for understanding sleep patterns in adolescents by connecting health outcomes to demographic and structural factors.^12^ For example, White adolescents have longer sleep duration compared to non-White adolescents, and adolescents in higher-income households report longer sleep duration compared to those in low-income families.^13–15^ However, these relationships between race and socioeconomic status may be more complex than they appear. The theory of Marginalization-related Diminished Returns (MDR) suggests that marginalized groups, particularly Black adolescents, may receive fewer health benefits from socioeconomic resources due to systemic barriers and structural racism.^16^ In one study of early adolescents aged 9–10 years, the protective effect of parental education on children’s sleep problems was diminished for non-Latino Black and Latino families compared to non-Latino White families,^17^ a pattern consistent with MDR observed in other health domains. Assessing whether these patterns apply to other dimensions of sleep in the present study can help clarify the extent to which socioeconomic advantage offers equal protective effects across racial and ethnic groups.

Beyond sleep duration and problems, adolescent research on disparities in other sleep dimensions, such as sleep timing and social jet lag, remains mixed.^18,19^ Additionally, socioeconomic stressors, such as income and parental education, may disrupt adolescent sleep by shaping neighborhood, household, and environmental factors.^20^ Understanding the relationship between sociodemographic factors and multidimensional sleep outcomes in adolescence is crucial for identifying targeted public health interventions to improve sleep health in vulnerable adolescent populations.^4^

Despite these implications, there is a paucity of data on the sociodemographic associations of irregular sleep patterns, such as chronotype and social jet lag, in early adolescence. Using a demographically diverse sample of early adolescents in the United States (US), this study aims to address this gap by (1) characterizing sleep duration, chronotype, and social jet lag and (2) examining how sociodemographic factors, including race and ethnicity, socioeconomic status, and sexual orientation relate to these sleep characteristics, and (3) exploring potential interactions between race and socioeconomic status in relation to sleep characteristics. To capture sleep irregularity, the Munich Chronotype Questionnaire (MCTQ) offers a nuanced understanding of sleep patterns and variability by measuring bedtime, wake time, and weekday-weekend differences, rather than focusing solely on duration.^21^ A better understanding of these patterns can inform interventions designed to reduce future health disparities.

## Methods

We performed cross-sectional analyses using data from the Adolescent Brain Cognitive Development (ABCD) Study (5.1 release). The ABCD Study is the largest longitudinal study of adolescent health, brain, and cognitive development in 11,875 children recruited from 21 sites across the US starting in 2016–2018. The ABCD Study sample, recruitment, protocol, and measures have previously been described in detail.^22^ The study was approved by the Institutional Review Board at the University of California, San Diego. Informed consent and assent were obtained from primary caregivers and participants, respectively.

### Sociodemographic factors

Participant age, sex (female or male), sexual orientation (heterosexual, gay/bisexual, maybe gay/bisexual, don’t understand the question, decline to answer), race and ethnicity (Asian, Black, Latino/Hispanic, Native American, White, other), household income (six categories), and parental education status (high school education or less vs. college education or more) were self-reported by the participants and/or their caregivers. Sex, race, and ethnicity were collected at baseline, while age, sexual orientation, household income, and parental education were collected at Year 3.

### Sleep - the Munich Chronotype Questionnaire

The MCTQ is a 17-item self-report tool that assesses sleep patterns and circadian preferences by collecting information on sleep timing, including bedtimes and wake times, across both school days and free days.^23^ To reflect the routines of the school-aged population in this study, weekdays are referred to as “school days,” while weekends and other non-school days are referred to as “free days.”^4^

#### Sleep duration and efficiency

For both school days and free days, sleep duration was calculated using MCTQ responses on bedtime, sleep latency, and wake time.^4^ Average weekly sleep duration was calculated as the weighted average of school day and free day sleep duration: [(school day sleep duration × school days per week) + (free day sleep duration × free days per week)]/7.^4^ For quality control, average sleep duration <3 h or >15 h were winsorized at 3 h (minimum) and 15 h (maximum) to retain all participants while minimizing outlier influence, adapted from cleaning methods from prior research.^24^ Derived from sleep duration, sleep efficiency represents the percent of time asleep in bed, calculated as (sleep duration / time in bed) × 100 for both school and free days, following established actigraphy protocols.^25^ Average weekly sleep efficiency was calculated as the weighted average of school day and free day sleep efficiency. Sleep efficiency values >100% or <50% were excluded as unrealistic (701 observations removed), consistent with data cleaning approaches that exclude non-sleep activities in time-in-bed calculations.^26^

The prevalence of participants meeting the American Academy of Sleep Medicine’s (AASM) age-specific sleep duration guidelines was calculated based on participants’ ages.^27^ According to the AASM, children aged 6–12 years are recommended to obtain 9–12 h of sleep per night, while adolescents aged 13–18 years are recommended to obtain 8–10 h per night.^27^ Our sample included participants aged 11–14 years, with the majority falling within the 12–13-year-old range. Therefore, we applied the 6–12-year-old guideline (9–12 h) to participants aged 11–12, and the 13–18-year-old guideline (8–10 h) to those aged 13–14.

#### Chronotype

The MCTQ calculates chronotype based on the midpoint of sleep on free days, representing an individual’s preferred timing of sleep and biological circadian rhythm preferences.^23^ Scores are continuous and range from 16 to 40, with higher scores indicating a tendency toward later sleep and wake times.^10^ Scores are measured on a 24 h clock in decimal form, where each unit represents an hour.^10^ For example, a score of 16 corresponds to a sleep midpoint at 4:00 p.m. A score of 25.33 means the midpoint is at 1:20 a.m. the next day (because 25.33 h is 1 h and 20 min past midnight).^10^ The highest possible score, 40, represents a sleep midpoint of 4:00 p.m. on the following day, indicating a very delayed sleep schedule.^10^ Individuals who had an alarm set on free days (1479 participants) were excluded from the MCTQ calculation, and participants with a chronotype score below the minimum cutoff of 16 (2436 participants) were excluded from the analysis.

#### Social jet lag

Absolute social jet lag, a continuous variable derived from the MCTQ, was calculated as the absolute difference in hours between sleep midpoint on school days and free days, reflecting the misalignment between social and biological bedtime preferences.^10,24^ This measure captures both bedtime and wake time differences between school and free days, as evidenced by the moderate correlations between social jet lag and wake time differences (*r* = 0.72) and bedtime differences (*r* = -0.37). To account for individual differences in chronotype, we used a sleep-corrected version of social jet lag,^10^ which more precisely captures misalignment between biological and social rhythms.^23^

### Statistical analyses

Using Stata 18 (StataCorp, College Station, TX), we conducted descriptive analyses to describe sleep characteristics and examine their sociodemographic associations in early adolescents. Key variables were summarized using means, standard deviations, and percentages. Survey-weighted multiple linear regression models were used to estimate associations between sociodemographic characteristics (age, sex, sexual orientation, race and ethnicity, household income, and parental education) and continuous sleep outcomes (sleep duration, chronotype, and absolute social jet lag) at the Year 3 follow-up, adjusting for study site and sociodemographic variables. Interaction with race/ethnicity and sleep outcomes by binary household income (<$75,000 vs. ≥$75,000) and parental education (high school education or less vs. college education or more) was analyzed.

Participants completed the MCTQ during the Year 3 follow-up (2019–2021). From this sample, we excluded 1854 participants missing sociodemographic data at baseline or Year 3 and 26 participants missing MCTQ sleep duration and social jet lag data, resulting in a sample size of 10,082 participants (Appendix A). Participants missing MCTQ chronotype data—specifically those who reported using an alarm on free days (*n* = 1479) and had a score below the minimum cutoff (*n* = 2436)—were retained in the main sample but excluded from analyses involving chronotype. Consequently, regression analyses for chronotype were conducted with a reduced sample size (*n* = 6167).

## Results

Of our adolescent sample (*N* = 10,082) at Year 3, the mean age was 12.9 (±0.7) years, 48.5% of the participants were female, 45.3% were participants from racial and ethnic minority groups, and 46.4% came from a household with an annual income of less than $75,000, the approximate median income in the US (Table 1). Participants had an average sleep duration of 8.9 h (±1.5), with 50.5% of 11–12-year-olds and 61.0% of 13–14-year-olds meeting sleep guidelines (Table 1).^27^ Adolescents had an average chronotype of 28.3 (±2.0), corresponding to a midpoint of sleep on free days at 4:13 a.m. (Table 1). Absolute social jet lag was 2.3 hours (±1.7), and 75.9% showed a difference of more than 1 hour of social jet lag between school days and free days (Table 1). Appendix B presents the average sleep duration and sleep efficiency across the week, on school days, and on free days.

**Table 1:** Sociodemographic and sleep characteristics of 10,082 Adolescent Brain Cognitive Development (ABCD) Study participants at Year 3 (2019–2021).

Sociodemographic and sleep characteristics	Mean (SD)/(%)
Age (years)	12.9 (0.7)
Sex	
Female	48.5%
Male	51.5%
Sexual orientation	
Heterosexual	82.3%
Gay/bisexual	8.9%
Maybe gay/bisexual	5.6%
Don’t understand the question	1.7%
Refuse to answer	1.6%
Race and ethnicity	
Asian	5.5%
Black	15.4%
Latino/Hispanic	19.8%
Native American	3.2%
Other	1.4%
White	54.7%
Household income	
$24,999 or less	15.0%
$25,000 to $49,999	16.4%
$50,000 to $74,999	15.0%
$75,000 to $99,999	14.8%
$100,000 to $199,999	28.2%
$200,000 or greater	10.7%
Parent’s highest education	
High school education or less	15.4%
College education or more	84.6%
Sleep duration	
Mean sleep duration (hours)	8.9 (1.5)
<8 h	22.5%
8–9 h	30.1%
9–10 h	30.5%
10–11 h	11.9%
11–12 h	2.8%
12+ h	2.2%
Satisfied sleep guidelines for 11–12 years^a^	50.5%
Satisfied sleep guidelines for 13–14 years^a^	61.0%
Chronotype^b^ (score, hh:mm)	28.3 (2.0), 4:13am
Social jet lag	
Mean social jet lag (hours)	2.3 (1.7)
≤1 h	24.1%
>1 h	75.9%

Sampling weights were applied to yield representative estimates based on the American Community Survey from the US Census. *SD* standard deviation.

^a^Sleep guidelines are based on the American Academy of Sleep guidelines for 6–12-year-olds (9–12 h) and 13–18-year-olds (8–10 h). The age range in our sample included 11–14-year-old adolescents, with most adolescents being 12–13 years old.

^b^Due to quality control purposes, the chronotype sample size was N = 6167.

Table 2 illustrates the multiple regression models examining the sociodemographic associations with average weekly sleep duration, chronotype, and absolute social jet lag among adolescents. **Appendices C** and **D** present the multiple regression models examining the sociodemographic associations with sleep duration (school days and free days) and sleep efficiency (average weekly, school days, and free days). Tables 3 and 4 show significant interactions and stratified race/ethnicity and sleep associations by binary household income (<$75,000 vs. ≥$75,000) and parental education (high school education or less vs. college education or more).

**Table 2:** Sociodemographic associations with sleep characteristics of 10,082 Adolescent Brain Cognitive Development (ABCD) Study participants at Year 3 (2019–2021).

	Sleep duration (hours)		Chronotype^a^ (score)		Social jet lag (hours)	
Sociodemographic characteristics	Adjusted B (95% CI)	p	Adjusted B (95% CI)	p	Adjusted B (95% CI)	p
Age	**-0.24 (-0.29, -0.19)**	**<0.001**	**0.26 (0.17, 0.34)**	**<0.001**	**0.19 (0.14, 0.25)**	**<0.001**
Sex						
Female	reference		reference		reference	
Male	0.01 (-0.06, 0.08)	0.791	0.04 (-0.08, 0.15)	0.549	-0.02 (-0.10, 0.05)	0.513
Sexual orientation						
Heterosexual	reference		reference			
Gay/bisexual	**-0.40 (-0.55, -0.26)**	**<0.001**	**0.71 (0.49, 0.92)**	**<0.001**	**0.37 (0.22, 0.52)**	**<0.001**
Maybe gay/bisexual	**-0.23 (-0.38, -0.09)**	**0.002**	**0.44 (0.20, 0.67)**	**<0.001**	0.17 (0.00, 0.34)	0.052
Don’t understand the question	**-0.43 (0.21, 0.65)**	**<0.001**	**-0.35 (-0.88, 0.19)**	**0.201**	**-0.52 (-0.76, - 0.28)**	**<0.001**
Refuse to answer	-0.13 (0.43, 0.17)	0.403	-0.14 (-0.57, 0.30)	0.538	0.03 (-0.25, 0.30)	0.855
Race and ethnicity						
Asian	0.00 (-0.12, 0.13)	0.953	-0.17 (-0.35, 0.02)	0.076	-0.09 (-0.22, 0.04)	0.197
Black	**-0.19 (-0.31, -0.07)**	**0.002**	**0.83 (0.60, 1.05)**	**<0.001**	**1.02 (0.89, 1.15)**	**<0.001**
Latino/Hispanic	**-0.15 (-0.28, -0.03)**	**0.012**	0.10 (-0.11, 0.31)	0.348	**0.28 (0.15, 0.41)**	**<0.001**
Native American	-0.03 (-0.25, 0.18)	0.762	**0.47 (0.02, 0.92)**	**0.041**	**0.66 (0.35, 0.96)**	**<0.001**
Other	-0.17 (-0.55, 0.21)	0.384	0.24 (-0.28, 0.76)	0.360	0.33 (-0.01, 0.67)	0.060
White	reference		reference		reference	
Household income						
$24,999 or less	-0.05 (-0.20, 0.10)	0.540	**0.61 (0.34, 0.87)**	**<0.001**	**0.58 (0.42, 0.75)**	**<0.001**
$25,000 to $49,999	-0.12 (-0.25, 0.01)	0.063	**0.49 (0.27, 0.71)**	**<0.001**	**0.48 (0.34, 0.62)**	**<0.001**
$50,000 to $74,999	**-0.16 (-0.27, -0.05)**	**0.006**	**0.38 (0.18, 0.57)**	**<0.001**	**0.33 (0.21, 0.46)**	**<0.001**
$75,000 to $99,999	-0.04 (-0.15, 0.06)	0.413	**0.16 (0.00, 0.32)**	**0.044**	**0.20 (0.09, 0.30)**	**<0.001**
$100,000 to $199,999	-0.02 (-0.10, 0.06)	0.591	-0.01 (-0.14, 0.12)	0.878	0.07 (-0.02, 0.16)	0.128
$200,000 or greater	reference		reference		reference	
Parent’s highest education						
High school education or less	-0.04 (-0.17, 0.09)	0.525	**0.26 (0.03, 0.50)**	**0.029**	**0.24 (0.10, 0.38)**	**0.001**
College education or more	reference		reference		reference	

**Bold** indicates *p* < 0.05. B = coefficient from linear regression model. Models represent the abbreviated output from the linear regression models with adjustment for Year 3 age, sex, sexual orientation, race/ethnicity, household income, parental education, and study site. Sampling weights were applied to yield representative estimates based on the American Community Survey from the US Census.

^a^Due to quality control purposes, the chronotype sample size was *N* = 6167.

**Table 3:** Race and ethnicity associations with average sleep duration stratified by household income in the Adolescent Brain Cognitive Development (ABCD) Study in Year 3 (N = 10,082).

	Sleep duration (hours)		
Race and ethnicity	B (95% CI)	p	p for interaction
<$75,000			
Asian	---	---	---
Black	-0.11(-0.28, 0.06)	0.192	**0.009**
Latino/Hispanic	---	---	---
Native American	---	---	---
Other	---	---	---
White	reference		
≥$75,000			
Asian	---	---	---
Black	**-0.35 (-0.52, -0.18)**	**<0.001**	**0.009**
Latino/Hispanic	---	---	---
Native American	---	---	---
Other	---	---	---
White	reference		

**Bold** indicates *p* < 0.05. B = coefficient from linear regression model. --- indicates nonsignificant interaction. Models represent the abbreviated output from the linear regression models including adjustment for age, sex, sexual orientation, parental education, and study site. Sampling weights were applied to yield representative estimates based on the American Community Survey from the US Census.

**Table 4:** Race and ethnicity associations with sleep characteristics stratified by parental education in the Adolescent Brain Cognitive Development (ABCD) Study in Year 3 (N = 10,082).

	Sleep duration (hours)			Chronotype^a^ (score)			Social jet lag (hours)		
Race and ethnicity	B (95% CI)	p	p for interaction	B (95% CI)	p	p for interaction	B (95% CI)	p	p for interaction
High school or less									
Asian	---	---	---	-0.77 (-1.60, 0.06)	0.068	**0.012**	---	---	---
Black	**0.60 (0.24, 0.95)**	**0.001**	**<0.001**	0.30 (-0.56, 1.17)	0.495	**0.045**	---	---	---
Latino / Hispanic	**0.41 (0.05, 0.76)**	**0.027**	**<0.001**	---	---	---	-0.04 (-0.43, 0.36)	0.857	**0.010**
Native American	**1.09 (0.46, 1.71)**	**0.001**	**<0.001**	---	---	---	---	---	---
Other	---	---	---	---	---	---	---	---	---
White	reference			reference			reference		
College or more									
Asian	---	---	---	-0.10 (-0.28, 0.09)	0.300	**0.012**	---	---	---
Black	**-0.34 (-0.47, -0.21)**	**<0.001**	**<0.001**	**0.96 (0.72, 1.20)**	**<0.001**	**0.045**	---	---	---
Latino / Hispanic	**-0.20 (-0.33, -0.07)**	**0.002**	**<0.001**	---	---	---	**0.37 (0.23, 0.52)**	**<0.001**	**0.010**
Native American	**-0.23 (-0.45, -0.01)**	**0.044**	**<0.001**	---	---	---	---	---	---
Other	---	---	---	---	---	---	---	---	---
White	reference			reference			reference		

**Bold** indicates p < 0.05. B = coefficient from linear regression model. --- indicates nonsignificant interaction. Models represent the abbreviated output from the linear regression models including adjustment for age, sex, sexual orientation, household income, and study site. Sampling weights were applied to yield representative estimates based on the American Community Survey from the US Census.

^a^Due to quality control purposes, the chronotype sample size was *N* = 6167.

### Age

Older age was associated with shorter sleep duration (coefficient [B] = -0.24; 95% confidence interval [CI] -0.29, -0.19; *p* < 0.001), later chronotype (B = 0.26; 95% CI 0.17, 0.34; *p* < 0.001), and greater absolute social jet lag (B = 0.19; 95% CI, 0.14, 0.25; *p* < 0.001) (Table 2).

### Sex

There were no significant associations between sex assigned at birth and sleep characteristics. However, sex differences were observed when examining school day and weekend patterns separately: males had longer school day sleep duration, but shorter free day sleep duration compared to females (Appendix C).

### Sexual orientation

Adolescents who self-identified as gay or bisexual had shorter sleep duration (B = -0.40; 95% CI -0.55, -0.26; *p* < 0.001), later chronotype (B = 0.71; 95% CI 0.49, 0.92; *p* < 0.001), and greater absolute social jet lag (B = 0.37; 95% CI 0.22, 0.52; *p* < 0.001) compared to their heterosexual peers. Adolescents who self-identified as “maybe” gay or bisexual had shorter sleep duration (B = -0.23; 95% CI -0.38, -0.09; *p* = 0.002) and later chronotype (B = 0.44; 95% CI 0.20, 0.67; *p* < 0.001) compared to their heterosexual peers (Table 2).

### Race and ethnicity

Black adolescents had shorter sleep duration (B = -0.19; 95% CI -0.31, -0.07; *p* = 0.002), later chronotype (B = 0.83; 95% CI 0.60, 1.05; *p* < 0.001), and greater absolute social jet lag (B = 1.02; 95% CI 0.89, 1.15; *p* < 0.001) compared to White adolescents. Latino/Hispanic adolescents had shorter sleep duration (B = -0.15; 95% CI -0.28, -0.03; *p* = 0.012) and greater absolute social jet lag (B = 0.28; 95% CI 0.15, 0.41; *p* < 0.001) compared to White adolescents. Native American adolescents had later chronotype (B = 0.47; 95% CI 0.02, 0.92; *p* = 0.041), and greater absolute social jet lag (B = 0.66; 95% CI 0.35, 0.96; *p* < 0.001) compared to White adolescents (Table 2).

### Household income

Adolescents from households earning $50,000 to $74,999 showed shorter sleep duration (B = -0.16; 95% CI -0.27, -0.05; *p* = 0.006) compared to those earning $200,000 or greater. Households earning less than $100,000 were associated with later chronotype and greater social jet lag compared to those earning $200,000 or greater **(**Table 2**)**. Lower income was associated with lower sleep efficiency (Appendix D).

### Parental education

Adolescents whose parents had high school education or less showed no difference in sleep duration but had later chronotype (B = 0.26; 95% CI 0.03, 0.50; *p* = 0.029) and greater absolute social jet lag (B = 0.24; 95% CI 0.10, 0.38; *p* = 0.001) compared to participants whose parents had college education or more.

### Race and ethnicity interactions with household income and parental education

The association between Black race and sleep duration significantly varied by household income (interaction *p* = 0.009). Among adolescents from households earning more than $75,000, Black race was associated with lower sleep duration (B = -0.35; 95% CI -0.52, -0.18; *p* < 0.001) compared to White race, while the association was nonsignificant for adolescents from households earning less than $75,000 (Table 3).

Parental education significantly modified associations between race/ethnicity and sleep duration for Black, Latino/Hispanic, and Native American adolescents (all three groups interaction *p* < 0.001). Among those with parents with a high school education or less, these groups showed longer sleep duration compared to White race; among those with college-educated parents, they showed shorter sleep duration compared to White race. The association between Asian (interaction *p* = 0.012) and Black race (interaction *p* = 0.045) and chronotype significantly differed by parental education. Asian was not significantly associated with chronotype at either level of parental education, but the strength of association varied. For adolescents with college-educated parents, Black race was associated with a later chronotype (B = 0.96; 95% CI: 0.72, 1.20; *p* < 0.001) compared to White race, while the association was nonsignificant for those with lower parental education. The association between Latino/Hispanic race/ethnicity and social jet lag significantly differed by parental education (interaction *p* = 0.010). Compared to White race, Latino/Hispanic race/ethnicity was associated with greater social jet lag only among adolescents with college-educated parents (B = 0.37; 95% CI: 0.23, 0.52; *p* < 0.001) (Table 4).

## Discussion

In a demographically diverse sample of early adolescents across the US (*N* = 10,082), several sociodemographic factors, including age, sexual orientation, race and ethnicity, household income, and parental education, were associated with various sleep measures, including sleep duration, chronotype, and social jet lag.^27,28^

### Sleep characteristics

Adequate sleep duration is crucial for adolescent development, as insufficient sleep duration is associated with impaired neurocognitive development and poorer academic performance.^29,30^ In our sample, adolescents had an average sleep duration of 8.9 hours, with 50.5% of 11–12-year-olds and 61.0% of 13–14-year-olds in our sample meeting the recommended sleep guidelines.^27^ Despite generally sufficient sleep duration in our sample, the average chronotype was 28.3, corresponding to a sleep midpoint of approximately 4:13 a.m. on free days. Previous ABCD data reported a similar average chronotype (27.8) at the Year 2 follow-up.^11^

In our study, average social jet lag was 2.3 h, with 75.9% of adolescents experiencing more than one hour, which is substantially higher than the 1.38 h mean reported in a prior ABCD study using pre-pandemic data.^11^ One possible explanation for this discrepancy may be the timing of data collection (2019–2021), which included early stages of the COVID-19 pandemic. Although remote learning may have temporarily alleviated early school start constraints, our sample likely reflects the return to in-person or hybrid instruction period, reintroducing weekday-weekend misalignment, particularly among those with later chronotypes.^9^ Additional contributing factors may include shorter lockdown durations, varied school reopening timelines, and a return to earlier weekday routines, possibly amplifying misalignment compared to the uniform pre-pandemic period. Regardless, a reported social jet lag of over one hour is associated with adverse sleep and health outcomes,^9^ and our observed mean of 2.3 h underscores the relevance of social jet lag as a public health concern.

### Age

Older age was associated with shorter average weekly sleep duration, later chronotypes, and greater absolute social jet lag. These findings align with prior research documenting age-related declines in sleep duration and emerging sleep irregularity during early adolescence.^14,31^ Shorter sleep among older adolescents may also be influenced by behavioral and environmental factors, such as greater problematic screen use,^32^ increased exposure to mature media content, and heightened academic and social demands.^33^ As adolescents age, the prevalence of mental health outcomes, including anxiety and depression, also rises,^34,35^ and these conditions have been associated with insomnia and irregular sleep patterns.^36^ Together, these factors may explain the age-related differences in sleep outcomes found in older adolescents.

### Sex

No differences in sex were observed for *average* weekly sleep duration, chronotype, or social jet lag in our study. These findings align with previous studies showing minimal or no sex-based differences in chronotype during early adolescence.^37,38^ As adolescents mature, chronotype generally shifts later, and sex differences may emerge due to differing pubertal timing, often resulting in earlier chronotypes in males compared to females.^39^ However, when examining school day versus free day sleep duration separately, we found that male sex was significantly associated with longer sleep on school days and shorter sleep on free days compared to females. Similar patterns have been reported in prior research,^40^ which have been attributed to females demonstrating increased wake-time irregularity and a greater reliance on compensating for weekday sleep debt by extending sleep on weekends.^41,42^

### Sexual orientation

Adolescents who self-identify as gay or bisexual reported lower average weekly sleep duration and exhibited later chronotype and greater social jet lag. While prior research has documented reduced sleep duration, longer sleep latency, and greater sleep disturbance in sexual minority youth,^12,13^ our study is among the first to link sexual orientation to later chronotype and greater social jet lag, further contributing to the growing body of evidence of sleep disparities affecting sexual minority adolescents. These findings are concerning given prior research linking poor sleep to increased risk of cardiovascular disease,^43^ for which sexual minority individuals are already at elevated risk.^44^ These findings also illuminate a broader disparity in cardiovascular health among sexual minority youth. The Minority Stress Theory provides a framework for understanding these differences, suggesting that discrimination and stigma experienced by minoritized groups can cause stress, negatively impacting sleep patterns and overall health.^45,46^ Sexual minority youth also have higher rates of bedtime screen use than non-sexual minority youth, which may worsen sleep outcomes.^47^

### Race and ethnicity

Black and Latino/Hispanic adolescents experienced shorter average weekly sleep durations compared to their White peers. These findings align with past research indicating that Black and Latino children have lower sleep duration than White children.^48^ Black and Native American adolescents exhibited later chronotypes compared to their White peers. Additionally, identifying as Black, Latino/Hispanic, or Native American was associated with higher absolute social jet lag. These findings are consistent with earlier research showing higher levels of social jet lag among non-Hispanic Black and Mexican American adolescents compared to non-Hispanic White adolescents.^31^ Additionally, our findings for Native American adolescents are especially noteworthy since they are a demographic that has been underrepresented in sleep and health research.^49^

### Household income and parental education

Lower household income and parental education were associated with later chronotypes and greater social jet lag. This is consistent with prior research linking lower household income to more severe insomnia symptoms^50^ and highlighting the role of economic hardship in disrupting sleep duration and quality in adolescents.^20^ Both material factors, unstable housing or limited access to resources, and subjective perceptions of financial strain have been independently associated with poorer sleep outcomes in youth.^20^ Lower household income was also associated with lower sleep efficiency, despite not being directly linked to shorter sleep duration. This suggests that while adolescents from lower-income households may spend a similar amount of time asleep as their higher-income peers, they are spending a lower proportion of time asleep in bed, potentially due to sleep disturbances. These findings align with recent research reporting reduced sleep efficiency among adolescents from lower-income households.^13^

Adolescents from lower-income households are more likely to be exposed to environmental and psychosocial stressors, including crowded living conditions, inconsistent schedules, and reduced access to quiet and structured sleep environments, all of which can disrupt sleep timing, sleep quality and contribute to larger discrepancies between school night and weekend sleep schedules.^13,51–53^

### Intersection of race and socioeconomic status

The most notable finding from the interaction and stratified analyses was that among adolescents from higher-income and higher parental education households, Black race was associated with poorer sleep outcomes. This finding aligns with the MDR theory, which posits that Black adolescents receive fewer health benefits from socioeconomic resources compared to their White peers due to structural racism and systemic barriers.^16^ Applying this framework, even at comparable levels of income and parental education, Black adolescents may experience poorer sleep due to factors, such as neighborhood disadvantage, discrimination, and environmental stressors that disproportionately affect them, undermining the protective effects of socioeconomic advantage. These findings suggest that improvements in household income and parental education alone may be insufficient to eliminate racial disparities in sleep health, highlighting the need for multi-level interventions that address both psychosocial and structural determinants of sleep.

### Limitations

The study faced several limitations. The underrepresentation of racially and ethnically minoritized adolescents within the study sample may limit external validity, and the use of self-reported sleep data introduces potential recall and reporting bias. Prior studies comparing MCTQ self-reports with objective measures, such as actigraphy or Fitbit have shown mixed validity across sleep variables.^54,55^ For instance, MCTQ-reported sleep duration and social jet lag show weak to moderate correlations with Fitbit data, with adolescents tending to overestimate sleep duration and social jet lag.^55^ In contrast, chronotype has shown a strong correlation with actigraphy-derived estimates in adult samples, though this finding may not fully generalize to adolescents.^54^ Overall, these patterns suggest that reporting bias is most pronounced for sleep duration and social jet lag, with adolescents likely reporting idealized or socially desirable sleep duration and inaccurately recalling weekday and weekend sleep timing. Another limitation is that adolescents who set their alarm on free days and had a chronotype below the minimum score of 16 were excluded from the chronotype variable, which reduced the available sample size for analyses involving this measure and may limit the power of chronotype findings.

### Implications and applications

This study highlights important clinical and policy implications for improving adolescent sleep health through targeted interventions. School-based policies, such as delaying start times and incorporating sleep education, have also shown promise in improving sleep duration and developmental outcomes when paired with structural changes.^4,56,57^ Integrating sleep education with these changes can reinforce healthy behaviors and help reduce disparities. Tailored interventions are especially important for vulnerable youth, as early school start times and limited access to sleep-supportive environments disproportionately affect marginalized groups, including those from lower-income households, racially and ethnically minoritized adolescents, and sexual minority adolescents who are more likely to face environmental and psychological barriers to healthy sleep.^58^ While this study examined the interaction between race and socioeconomic status, future research should extend this intersectional approach to include other identities, such as sexual orientation and gender.

In addition to clinical and policy approaches, parental involvement plays a vital role in supporting adolescent sleep. Parental monitoring, particularly regarding limiting screen use before bed, has been linked to earlier sleep onset and longer sleep duration.^4,59,60^ Interventions that involve parents in setting consistent sleep-wake routines and reducing evening device use have shown promise in mitigating sleep disruptions.^61^ Educational programs that equip parents to model and reinforce good sleep hygiene can improve adolescent sleep and support broader emotional and behavioral outcomes.^61^

## Conclusion

This study reveals key sociodemographic disparities in sleep patterns among a diverse, national sample of adolescents (mostly 12–13 years) in the ABCD Study. Specifically, older age, gay/bisexual sexual orientation, Black, Latino/Hispanic, Native American, lower household income, and lower parental education were associated with poorer sleep outcomes. Among higher-income or higher-education households, Black adolescents reported worse sleep outcomes than their White counterparts, supporting the MDR theory. These findings underscore the need for targeted interventions to reduce sleep disparities, which may contribute to various adverse health outcomes. Investigating modifiable behaviors, including bedtime screen use and daily physical activity, could offer valuable insight into both risk and protective factors influencing adolescent sleep health.

## Supplementary information


Supplementary Material.


## Data Availability

Data used in the preparation of this article were obtained from the ABCD Study (https://abcdstudy.org), held in the NIH Brain Development Cohorts (NBDC) Portal.
