# Supplementary material for: Social epidemiology of multidimensional sleep health in early adolescence
Source: Pediatr Res. 2025 Nov 28;100(1):176–84. doi: 10.1038/s41390-025-04616-7 (PMC12772439; doi:10.1038/s41390-025-04616-7)
Supplement: Supplementary file 1 [file 41390_2025_4616_MOESM1_ESM.pdf]

Appendix A. Comparison of the sociodemographic characteristics of the Adolescent Brain Cognitive Development (ABCD) Study participants included vs. excluded in the analysis

Sociodemographic characteristics	Included (n=10,082)	Excluded (n=1,880)	p
Age (years)	12.9 (0.7)	13.0 (0.7)	0.121
Sex			0.176
Female	48.5%	50.5%	
Male	51.5%	49.5%	
Sexual orientation			**<0.001**
Heterosexual	82.3%	83.9%	
Gay/bisexual	8.9%	4.8%	
Maybe gay/bisexual	5.6%	2.3%	
Don't understand the question	1.7%	7.2%	
Refuse to answer	1.6%	1.7%	
Race and ethnicity			**<0.001**
Asian	5.5%	5.6%	
Black	15.4%	27.9%	
Latino/Hispanic	19.8%	21.7%	
Native American	3.2%	3.3%	
Other	1.4%	1.7%	
White	54.7%	39.8%	
Household income			**<0.001**
$24,999 or less	15.0%	24.6%	
$25,000 to $49,999	16.4%	22.1%	
$50,000 to $74,999	15.0%	14.6%	
$75,000 to $99,999	14.8%	8.8%	
$100,000 to $199,999	28.2%	24.2%	
$200,000 or greater	10.7%	5.8%	
Parent's highest education			**<0.001**
High school education or less	15.4%	25.7%	
College education or more	84.6%	74.3%	

Appendix B. Sleep characteristics (weekly, school day, free day) of 10,082 Adolescent Brain Cognitive Development (ABCD) Study participants at Year 3 (2019–2021)

Sleep characteristics	Mean (SD)
Sleep duration (hours)	
Weekly sleep duration	8.9 (1.5)
School day sleep duration	8.7 (1.7)
Free day sleep duration	9.7 (2.3)
Sleep efficiency[a] (%)	
Weekly sleep efficiency	94.1 (6.8)
School day sleep efficiency	94.1 (6.9)
Free day sleep efficiency	94.8 (6.9)

Sampling weights were applied to yield representative estimates based on the American Community Survey from the US Census. SD = standard deviation

[a] Due to quality control purposes, the sleep efficiency sample size was N=9,043

Appendix C. Sociodemographic associations with sleep duration (school days, free days) of 10,082 Adolescent Brain Cognitive Development (ABCD) Study participants at Year 3 (2019–2021)

	Sleep duration, school days (hours)		Sleep duration, free days (hours)	
Sociodemographic characteristics	Adjusted B (95% CI)	p	Adjusted B (95% CI)	p
Age	**-0.30 (-0.36, -0.25)**	**<0.001**	-0.08 (-0.15, -0.02)	**0.016**
Sex				
Female	reference		reference	
Male	**0.11 (0.04, 0.18)**	**0.003**	**-0.23 (-0.32, -0.14)**	**<0.001**
Sexual orientation				
Heterosexual	reference		reference	
Gay/bisexual	**-0.47 (-0.63, -0.32)**	**<0.001**	**-0.26 (-0.44, -0.08)**	**0.006**
Maybe gay/bisexual	**-0.24 (-0.40, -0.08)**	**0.003**	-0.12 (-0.29, 0.06)	0.186
Don't understand the question	**0.50 (0.25, 0.74)**	**<0.001**	**0.35 (0.04, 0.65)**	**0.025**
Refuse to answer	-0.14 (-0.47, 0.18)	0.391	0.09 (-0.31, 0.50)	0.649
Race and ethnicity				
Asian	0.09 (-0.04, 0.21)	0.189	-0.01 (-0.16, 0.15)	0.948
Black	**-0.31 (-0.44, -0.19)**	**<0.001**	**-0.19 (-0.35, -0.03)**	**0.019**
Latino/Hispanic	**-0.15 (-0.25, -0.04)**	**0.007**	-0.03 (-0.17, 0.11)	0.659
Native American	-0.05 (-0.27, 0.17)	0.633	0.18 (-0.12, 0.46)	0.259
Other	-0.16 (-0.57, 0.26)	0.460	-0.04 (-0.50, 0.42)	0.854
White	reference		reference	
Household income				
$24,999 or less	-0.04 (-0.20, 0.12)	0.615	**-0.20 (-0.39, -0.01)**	**0.041**
$25,000 to $49,999	-0.13 (-0.27, 0.00)	0.052	-0.15 (-0.31, 0.02)	0.083
$50,000 to $74,999	**-0.16 (-0.27, -0.04)**	**0.010**	**-0.19 (-0.34, -0.04)**	**0.012**
$75,000 to $99,999	0.01 (-0.10, 0.12)	0.859	**-0.18 (-0.32, -0.05)**	**0.009**
$100,000 to $199,999	-0.01 (-0.10, 0.08)	0.801	**-0.11 (-0.21, -0.01)**	**0.031**
$200,000 or greater	reference		reference	
Parent's highest education				
High school education or less	-0.03 (-0.17, 0.11)	0.697	-0.04 (-0.21, 0.14)	0.675
College education or more	reference		reference	

Bold indicates p<0.05. B=coefficient from linear regression model. Models represent the abbreviated output from the linear regression models with adjustment for Year 3 age, sex, sexual orientation, race/ethnicity, household income, parental education, and study site. Sampling weights were applied to yield representative estimates based on the American Community Survey from the US Census.

Appendix D. Sociodemographic associations with sleep efficiency (weekly, school days, free days) of 9,043 Adolescent Brain Cognitive Development (ABCD) Study participants at Year 3 (2019–2021)

Sociodemographic characteristics	Sleep efficiency, weekly (%)		Sleep efficiency, school days (%)		Sleep efficiency, free days (%)	
	Adjusted B (95% CI)	p	Adjusted B (95% CI)	p	Adjusted B (95% CI)	p
Age	-0.22 (-0.45, 0.02)	0.068	-0.22 (-0.48, 0.05)	0.112	-0.22 (-0.48, 0.04)	0.101
Sex						
Female	reference		reference		reference	
Male	0.27 (-0.04, 0.58)	0.090	0.27 (-0.07, 0.62)	0.122	**0.43 (0.08, 0.78)**	**0.015**
Sexual orientation						
Heterosexual	reference		reference		reference	
Gay/bisexual	**-2.38 (-3.11, -1.66)**	**<0.001**	**-2.58 (-3.38, -1.78)**	**<0.001**	**-1.95 (-2.75, -1.15)**	**<0.001**
Maybe gay/bisexual	**-1.61 (-2.34, -0.87)**	**<0.001**	**-1.71 (-2.53, -0.89)**	**<0.001**	**-1.10 (-1.91, -0.28)**	**0.008**
Don't understand the question	0.25 (-0.65, 1.14)	0.589	0.38 (-0.62, 1.39)	0.455	0.26 (-0.71, 1.23)	0.598
Refuse to answer	-0.60 (-1.81, 0.62)	0.338	-0.74 (-2.14, 0.66)	0.303	-0.24 (-1.52, 1.03)	0.707
Race and ethnicity						
Asian	**0.71 (0.08, 1.35)**	**0.028**	**0.90 (0.21, 1.59)**	**0.011**	0.53 (-0.15, 1.21)	0.126
Black	**-1.79 (-2.32, -1.26)**	**<0.001**	**-1.77 (-2.37, -1.16)**	**<0.001**	**-1.88 (-2.46, -1.29)**	**<0.001**
Latino/Hispanic	-0.36 (-0.83, 0.10)	0.128	-0.31 (-0.84, 0.23)	0.258	-0.47 (-1.01, 0.06)	0.081
Native American	-0.49 (-1.39, 0.41)	0.287	-0.42 (-1.44, 0.61)	0.426	-0.68 (-1.76, 0.40)	0.218
Other	-1.37 (-3.02, 0.29)	0.106	-1.31 (-3.08, 0.46)	0.146	-1.41 (-3.67, 0.85)	0.221
White	reference		reference		reference	
Household income						
$24,999 or less	**-1.27 (-1.98, -0.56)**	**<0.001**	**-1.36 (-2.17, -0.55)**	**0.001**	**-1.02 (-1.74, -0.29)**	**0.006**
$25,000 to $49,999	**-0.99 (-1.57, -0.42)**	**0.001**	**-1.17 (-1.82, -0.53)**	**<0.001**	**-1.03 (-1.67, -0.39)**	**0.002**
$50,000 to $74,999	**-1.24 (-1.77, -0.71)**	**<0.001**	**-1.29 (-1.88, -0.70)**	**<0.001**	**-1.13 (-1.73, -0.54)**	**<0.001**
$75,000 to $99,999	**-0.83 (-1.32, -0.35)**	**0.001**	**-0.70 (-1.23, -0.17)**	**0.009**	**-1.05 (-1.60, -0.50)**	**<0.001**
$100,000 to $199,999	**-0.65 (-1.02, -0.28)**	**0.001**	**-0.66 (-1.07, -0.25)**	**0.002**	**-0.74 (-1.16, -0.32)**	**<0.001**
$200,000 or greater	reference		reference		reference	
Parent's highest education						
High school education or less	-0.38 (-1.00, 0.25)	0.237	-0.49 (-1.20, 0.23)	0.181	-0.53 (-1.21, 0.15)	0.125
College education or more	reference		reference		reference	

Bold indicates p<0.05. B=coefficient from linear regression model. Models represent the abbreviated output from the linear regression models with adjustment for Year 3 age, sex, sexual orientation, race/ethnicity, household income, parental education, and study site. Sampling weights were applied to yield representative estimates based on the American Community Survey from the US Census.
